# Tau seeding activity in skin biopsy differentiates tauopathies from synucleinopathies

**DOI:** 10.1038/s41531-024-00728-9

**Published:** 2024-06-15

**Authors:** Ilaria Linda Dellarole, Elena Vacchi, Inigo Ruiz-Barrio, Sandra Pinton, Andrea Raimondi, Stefania Rossi, Sara Morandi, Giovanni Bianco, Merve Begum Bacinoglu, Annalisa Lombardo, Luigi Celauro, Claudio Staedler, Salvatore Galati, Javier Pagonabarraga, Jaime Kulisevsky, Giuseppe Legname, Claudio Gobbi, Alain Kaelin-Lang, Fabio Moda, Giorgia Melli

**Affiliations:** 1 Division of Neurology 5 and Neuropathology, Fondazione IRCCS Istituto Neurologico Carlo Besta, Milan, Italy; 2 Neurodegenerative Diseases Group, Laboratories for Translational Research, Ente Ospedaliero Cantonale, Bellinzona, Switzerland; 3 Faculty of Biomedical Sciences, Università della Svizzera Italiana, Lugano, Switzerland; 4 Movement Disorders Unit, Neurology Department, Hospital de la Santa Creu i Sant Pau, Barcelona, Spain; 5 Neurology Department, Neurocenter of Southern Switzerland, Ente Ospedaliero Cantonale, Lugano, Switzerland; 6 Institute for Research in Biomedicine, Università della Svizzera italiana, Bellinzona, Switzerland; 7 Laboratory of Prion Biology, Department of Neuroscience, Scuola Internazionale Superiore Di Studi Avanzati (SISSA), Trieste, Italy; 8 Department of Neurology, University Hospital of Basel, Basel, Switzerland; 9 Department of Neurology, Inselspital, Bern University Hospital, University of Bern, Bern, Switzerland

**Keywords:** Diagnostic markers, Neurodegenerative diseases

## Abstract

Most neurodegenerative diseases lack definitive diagnostic tests, and the identification of easily accessible and reliable biomarkers remains a critical unmet need. Since tau protein is highly expressed in skin of tauopathies patients, we aimed to exploit the ultrasensitive seeding activity assay (SAA) to assess tau seeding activity in skin of patients with tauopathies. In this multicentric, case-control study, patients with tauopathies and synucleinopathies were consecutively recruited and sex-matched to healthy controls (HC). Subjects underwent a double 3 mm skin biopsy in cervical area and ankle. Skin tau-SAA, using TauK18 and TauK19 as reaction substrates for 4R and 3R isoforms, seeding score, clinical scales, biochemical and morphological characterization of SAA end-products were evaluated. We analyzed 58 subjects: 24 tauopathies (18 progressive supranuclear palsy, PSP, and 6 corticobasal degeneration, CBD), 20 synucleinopathies (14 Parkinson’s disease, PD, and 6 multiple system atrophy, MSA), and 14 HC. PSP and CBD showed higher tau seeding activity at both anatomical sites. A greater sensitivity of 4R-SAA than 3R-SAA was observed. 4R tau-SAA identified tauopathies with 71% sensitivity and 93% specificity. Accuracy was higher for PSP than CBD: PSP vs HC / PD (AUC 0.825), while CBD vs HC / PD (AUC 0.797), and PSP vs MSA (AU 0.778). SAA end-products showed differences in biochemical and morphological characterization according to the anatomical site. Skin tau-SAA identifies tauopathies with good accuracy and can be used to implement the in-vivo clinical diagnosis of patients with neurodegenerative diseases. Further characterization of peripheral tau seed in skin may elucidate the structure of tau deposits in brain.

## Introduction

Tauopathies are the most common proteinopathies of the human nervous system and are characterized by the deposition of abnormal tau protein, such as neurofibrillary tangles and neuropil threads in nervous cells^1^. Currently, more than 26 different tauopathies are known, including Alzheimer’s disease (AD), progressive supranuclear palsy (PSP), corticobasal degeneration (CBD), Pick’s disease, chronic traumatic encephalopathy, tangle-only dementia, and frontotemporal dementia with parkinsonism linked to chromosome 17 (FTDP-17)^2–4^. In the adult human brain, six tau isoforms are expressed, resulting from the alternative splicing of MAPT gene transcripts^1^ on chromosome 17q21.31. Alternative splicing of exons 2 and 3 generates the isoforms 0N, 1N, and 2N, respectively, while alternative splicing of exon 10 generates isoforms containing 3 or 4 carboxy-terminal repeat domains, resulting in 3R or 4R isoforms^1^. In healthy conditions, the adult human brain contains similar amount of 3R and 4R tau, while in neurodegenerative diseases, this ratio is often altered: PSP and CBD tau aggregates are prevalently formed by 4R, while in AD by both 3R and 4R^5^.

Similar to α-synuclein and β-amyloid, misfolded tau can template the conformational conversion of normal monomers and propagate in the nervous system through specific spatio-temporal pathways^6^; this phenomenon is called seeding activity. In analogy to prion diseases, where various strains of prions (PrP^Sc^) contribute to the phenotypic heterogeneity of the disease, several strains of tau, each possessing distinct seeding and spreading abilities, have been described^7^. Pathological tau isolated from PSP brains was conformationally distinct from, and had greater seeding ability than, pathological tau isolated from AD brains^7^.

The current clinical diagnosis of tauopathies is challenging, especially in early phases, due to variants and overlaps in the clinical spectrum. The definitive diagnosis still relies on post-mortem neuropathological analysis based on the anatomical and cellular distributions of intracytoplasmic filamentous deposits of hyperphosphorylated tau^8^. The lack of more specific disease biomarkers hinders the possibility of developing effective disease-modifying therapies.

Recently developed ultrasensitive technologies called Seed Amplification Assays (SAAs), like the Real-Time Quaking-Induced Conversion assay (RT-QuIC) and Protein misfolding cyclic amplification (PMCA), have been developed in the field of prions diseases and allowed detection of prion in several peripheral fluids^9^. SAA are now being extended to the study of neurodegenerative diseases to detect minimal amount of misfolded proteins called seeds in CSF and other peripheral tissues (e.g., blood, skin, and olfactory mucosa)^10–12^. Selective assays for 4R^11^, 3R^13^, or combined 3R/4R^14^ tau strains detection by SAA have been developed in post-mortem brains and CSF, using fragments of recombinant tau proteins referred to as TauK18, for tau 4R, and TauK19, for tau 3R. These studies have further shown the distinct seeding activity of different tau strains^11,13,14^. Moreover, preliminary evidence in the field of synucleinopathies suggests that the amyloid fibrils generated by SAA can acquire biochemical and morphological features, which may be dictated by the α-synuclein strain and exploitable to discriminate diseases^10,12,15^. For this reason, SAA reaction products are currently being studied with several techniques, including Western blot after limited enzymatic digestions, transmission electron microscopy, protein-NMR, and Raman spectroscopy, among others^10,12,16,17^.

Due to its easy, non-invasive accessibility and its great content in nerve fibers, skin has emerged as a candidate for biomarker discovery in synucleinopathies. Phosphorylated, oligomeric α-synuclein, and a small fiber neuropathy have been detected in the skin of patients with Parkinson’s disease (PD) and multiple system atrophy (MSA)^18–21^.

We recently characterized tau protein in skin and showed that its concentration and amount of 4R tau isoform transcript level is higher in skin biopsy of tauopathies than healthy controls (HC) and synucleionopathies^22^; therefore, we hypothesized that we could exploit tau-SAA in skin samples of PSP and CBD and determine its diagnostic capacity vs PD and MSA.

## Results

### Demographic and clinical characteristics of study groups

A total of 116 skin samples from 58 living subjects were analyzed, namely 24 tauopathies (18 PSP and 6 CBD), 20 synucleinopathies (14 PD and 6 MSA), and 14 HC. Clinical characteristics of main groups are summarized in Table 1 and for sub-groups of disease in Table 2.

**Table 1:** Demographic and clinical characteristics of the three main groups of patients

Variable	HC (*n* = 14)	TAU (*n* = 24)	SYN (*n* = 20)	Overall *P*-Value	TAU vs HC	SYN vs HC	TAU vs SYN
Age (y)	59 ± 8	73 ± 8	66 ± 10	**0.000**	**0.000**	**0.050**	**0.012**
Sex (%M)	50.0%	62.5%	65.0%	0.661	0.339	0.301	0.558
Disease duration (y)	–	4 ± 2	4 ± 3	0.614	–	–	0.614
Age of onset (y)	–	69 ± 8	62 ± 10	**0.015**	–	–	**0.015**
H&Y	–	3.0 [2.0–4.0]	2.3 [1.0–3.0]	0.118	–	–	0.118
UPDRS-I	–	8.0 [6.0–9.0]	7.0 [3.0–11.5]	0.702	–	–	0.710
UPDRS-II	–	10.0 [3.0–26.0]	7.0 [3.0–13.5]	0.408	–	–	0.418
UPDRS-III	–	33.0 [21.0–51.0]	17.5 [10.0–27.5]	**0.008**	–	–	**0.008**
UPDRS-TOT	–	42.0 [25.0–61.0]	33.0 [17.5–50.0]	0.294	–	–	0.318
COMPASS-31_OH	–	0.0 [0.0–0.0]	6.0 [0.0–16.0]	**0.049**	–	–	0.099
COMPASS-31_VM	–	0.0 [0.0–0.0]	0.0 [0.0–0.0]	0.970	–	–	1.000
COMPASS-31_SM	–	4.0 [0.0–6.3]	3.2 [0.0–6.3]	0.964	–	–	0.965
COMPASS-31_GI	–	1.5 [0.0–4.0]	4.9 [0.2–6.9]	**0.032**	–	–	**0.035**
COMPASS-31_BL	–	2.0 [0.0–3.0]	0.0 [0.0–1.1]	0.128	–	–	0.149
COMPASS-31_PM	–	0.0 [0.0–2.0]	0.0 [0.0–2.0]	0.911	–	–	0.919
COMPASS-31_TOT	–	10.5 [4.7–18.8]	18.5 [6.3–30.6]	0.148	–	–	0.149
BDI-II	–	7.0 [5.0–15.0]	8.0 [4.0–14.0]	0.554	–	–	0.562
MMSE	–	26.5 [22.8–27.8]	29.0 [29.0–30.0]	**0.003**	–	–	**0.002**
MoCA	–	21.0 [17.3–22.8]	27.0 [25.0–28.0]	**0.005**	–	–	**0.004**
FAB	–	12.0 [8.0–14.5]	–	**–**	–	–	**–**
Smell test	–	9.0 [8.3–10.8]	6.0 [4.0–10.0]	0.092	–	–	0.095
RBDSQ	–	0.0 [0.0–2.0]	2.5 [1.0–5.0]	**0.012**	–	–	**0.012**
LEDD	–	400.0 [0.0–600.0]	288.0 [0.0–625.0]	0.945	–	–	1.957

Variables are reported as mean ± SD, median [interquartile range], and absolute number (percentage), as appropriate. A *P* < 0.05 was considered significant and shown in bold.

**Table 2:** Demographic and clinical characteristics of subgroups of patients based on the clinical diagnosis

Variable	HC (*n* = 14)	PSP (*n* = 18)	CBD (*n* = 6)	PD (*n* = 14)	MSA (*n* = 6)	Overall *P*-value	PSP vs HC	CBD vs HC	PD vs HC	MSA vs HC	PSP vs CBD	PSP vs PD	PSP vs MSA	CBD vs PD	CBD vs MSA	PD vs MSA
Age	59 ± 8	72 ± 7	74 ± 10	66 ± 10	64 ± 8	**0.001**	**0.000**	**0.020**	**0.044**	0.312	0.454	0.077	**0.040**	0.109	0.132	0.718
Sex (%M)	50.0%	66.7%	50.0%	71.4%	50.0%	0.702	0.278	0.685	0.220	0.686	0.397	0.541	0.397	0.336	0.716	0.397
Disease duration	–	4 ± 2	3 ± 1	4 ± 4	4 ± 3	0.778	–	–	–	–	1.000	0.408	0.759	0.579	0.699	0.639
Age of onset	–	69 ± 7	70 ± 11	63 ± 10	60 ± 8	0.095	–	–	–	–	0.431	0.133	**0.036**	0.087	0.065	0.467
H&Y	–	3.0 [3.0–4.0]	2.0 [1.8–3.3]	2.0 [1.0–3.0]	2.8 [1.9–5.0]	**0.043**	–	–	–	–	0.062	**0.021**	0.658	0.718	0.394	0.207
UPDRS-I	–	8.0 [6.0–9.0]	–	5.0 [2.5–10.5]	10.5 [7.0–16.2]	0.252	–	–	–	–	–	0.351	0.315	–	–	0.163
UPDRS-II	–	10.0 [3.0–26.0]	–	6.0 [3.0–11.0]	10.5 [3.3–14.8]	0.573	–	–	–	–	–	0.351	1.000	–	–	0.549
UPDRS-III	–	40.0 [21.0–51.5]	27.0 [15.3–41.5]	18.0 [7.0–23.5]	17.0 [12.5–48.5]	0.059	–	–	–	–	0.280	**0.003**	0.227	0.179	1.000	0.443
UPDRS-TOT	–	42.0 [25.0–61.0]	–	28.0 [14.5–50.0]	40.5 [24.0–70.5]	0.391	–	–	–	–	–	0.241	1.000	–	–	0.412
COMPASS-31_OH	–	0.0 [0.0–0.0]	0.0 [0.0–24.0]	6.0 [0.0–17.0]	6.0 [0.0–19.0]	0.208	–	–	–	–	0.574	0.102	0.235	0.859	0.905	0.968
COMPASS-31_TOT	–	11.0 [8.0–18.0]	5.0 [1.0–24.0]	15.4 [4.3–33.5]	19.2 [8.9–29.5]	0.482	–	–	–	–	0.738	0.331	0.235	0.432	0.381	0.659
BDI-II	–	7.0 [5.0–15.0]	–	5.0 [2.0–12.5]	13.0 [7.5–14.5]	0.234	–	–	–	–	–	0.250	0.438	–	–	0.156
MMSE	–	26.5 [22.8–27.8]	–	29.5 [28.8–30.0]	29.0 [28.0–30.0]	**0.010**	–	–	–	–	–	**0.004**	**0.045**	–	–	0.823
MoCA	–	21.0 [17.3–22.8]	–	27.0 [24.5–28.3]	27.0 [24.5–27.5]	**0.018**	–	–	–	–	–	**0.008**	**0.030**	–	–	0.823
FAB	–	9.0 [6.0–12.5]	14.5 [11.5–17.5]	–	–	0.065	–	–	–	–	0.063	–	–	–	–	–
Smell test	–	9.0 [8.3–10.8]	–	5.5 [3.0–9.0]	10.0 [6.0–11.5]	**0.045**	–	–	–	–	–	**0.020**	0.833	–	–	0.107
RBDSQ	–	1.0 [0.0–3.0]	0.0 [0.0–0.0]	3.0 [1.8–5.5]	1.5 [0.0–5.3]	**0.019**	–	–	–	–	0.081	**0.032**	0.609	**0.008**	0.114	0.353
LEDD	–	425.0 [103.5–600.0]	0.0 [0.0–350.0]	288.0 [50.0–750.0]	150.0 [0.0–537.5]	0.225	–	–	–	–	0.109	0.846	0.178	0.117	0.537	0.416

Variables are reported as mean ± SD, median [interquartile range], and absolute number (percentage), as appropriate. A *P* < 0.05 was considered significant and shown in bold.

Among groups, no significant differences were observed in sex ratio, disease duration, age of onset, and levodopa equivalent daily dose (LEDD). However, patients were older than HC, and PSP than MSA. In general, tauopathies showed a later onset, a more severe motor impairment by the Movement Disorder Society-Unified Parkinson’s Disease Rating Scale (MDS-UPDRS) part III, a higher cognitive impairment by the Montreal Cognitive Assessment (MoCA) and Mini-Mental State Evaluation (MMSE), and lower REM sleep behavior disorder (RBD) by the RBD screening questionnaire (RBDSQ) than synucleinopathies. In detail, PSP showed more severe disease gravity than PD by Hoehn and Yahr (H&Y) and UPDRS-III, a higher cognitive impairment vs PD and vs MSA by MMSE and MoCA, and a lower olfactory impairment than PD. Both PSP and CBD showed lower RBD symptoms than PD.

### Kinetics of TauK18- and TauK19-SAA reactions in skin

**Fig. 1:**
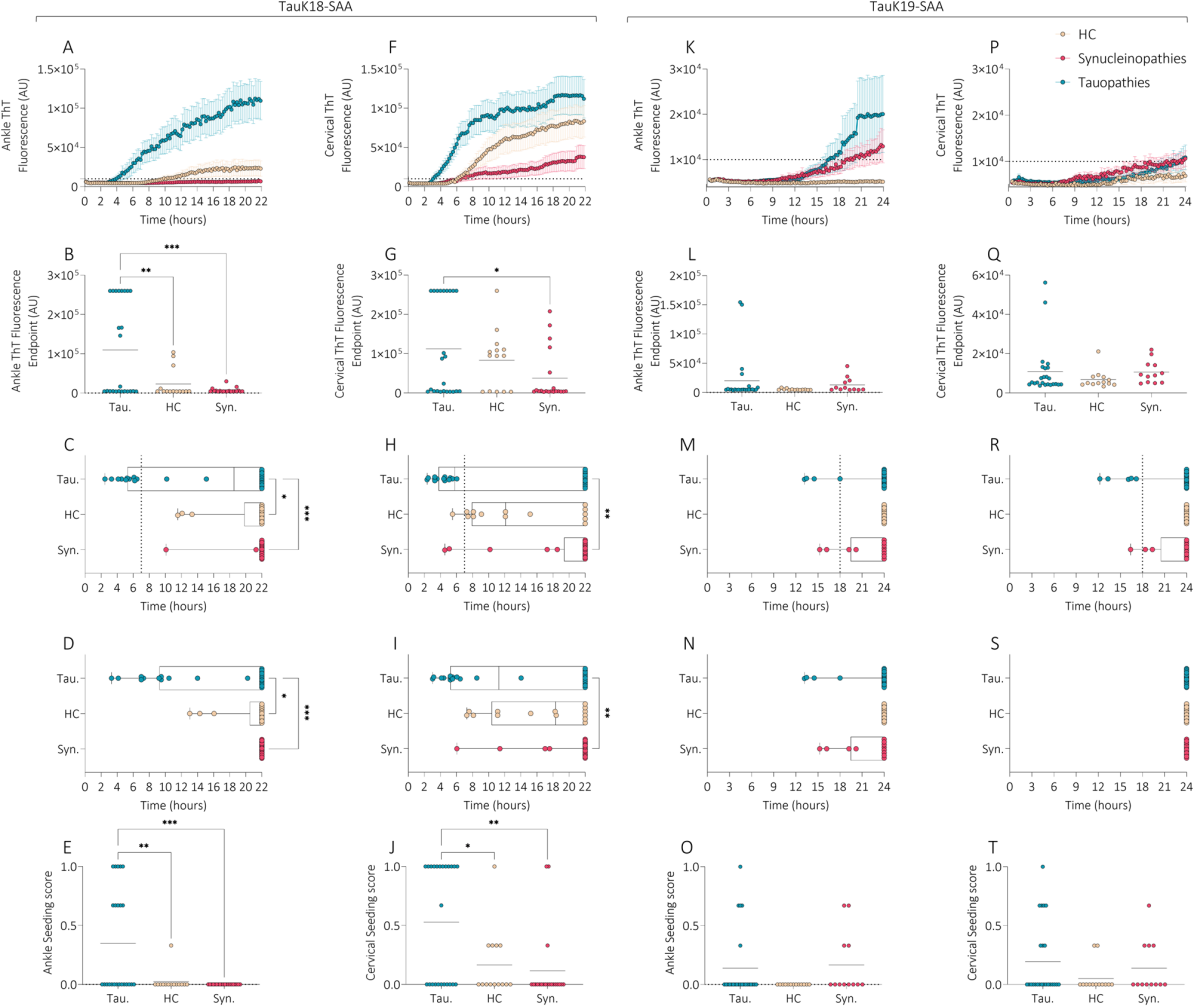
TauK18- and TauK19-SAA analyses at ankle and cervical sites in tauopathies, synucleinopathies, and healthy controls. Kinetic curves of TauK18 and TauK19 seeding activity in ankle (**A**, **K**) and cervical (**F**, **P**) sites, respectively. Each dot represents the mean (±SE) of the ThT fluorescence intensity of all samples tested per group against time. The dotted line indicates the selected threshold at 10.000 arbitrary units (AU). **B**, **G**, **L**, **Q** Scatter plot of final ThT fluorescence intensities: dots represent mean values of final ThT fluorescence intensity for individual patients and bars the mean of the group. **C**, **H**, **M**, **R** Box and Whisker Plot showing the Lag time defined as the reaction time required to pass the threshold of 10.000 AU. The dotted line shows the selected time points (7 h for TauK18 or 18 h for TauK19) to define the positive and negative subjects. **D**, **I**, **N**, **S** Box and Whisker Plot showing the T50, defined as the reaction time required to reach 50% of maximum fluorescence detected. **E**, **J**, **O**, **T** Seeding score defined as the number of replicates out of three that cross the threshold of 10.000 AU at 7 h. Each dot represents a patient and bars the mean of the group.

Samples of patients with tauopathies showed higher seeding activity than those of patients with synucleinopathies and HC at both anatomical sites. All kinetic measures of both TauK18-SAA and TauK19-SAA for main groups and sub-groups of disease are reported in Supplementary Tables 1 and 2. TauK18-SAA detected higher Thioflavin T (ThT) signal, reduced lag time (time to threshold), and T50 (time to 50% of maximum fluorescence) in tauopathies (Fig. 1A–T). Cervical skin showed higher ThT signal and shorter lag time than in ankle in HC and synucleinopathies, and a steeper kinetic curve in tauopathies. The seeding score was also significantly higher in tauopathies vs other groups and in cervical area vs ankle in all groups.

**Fig. 2:**
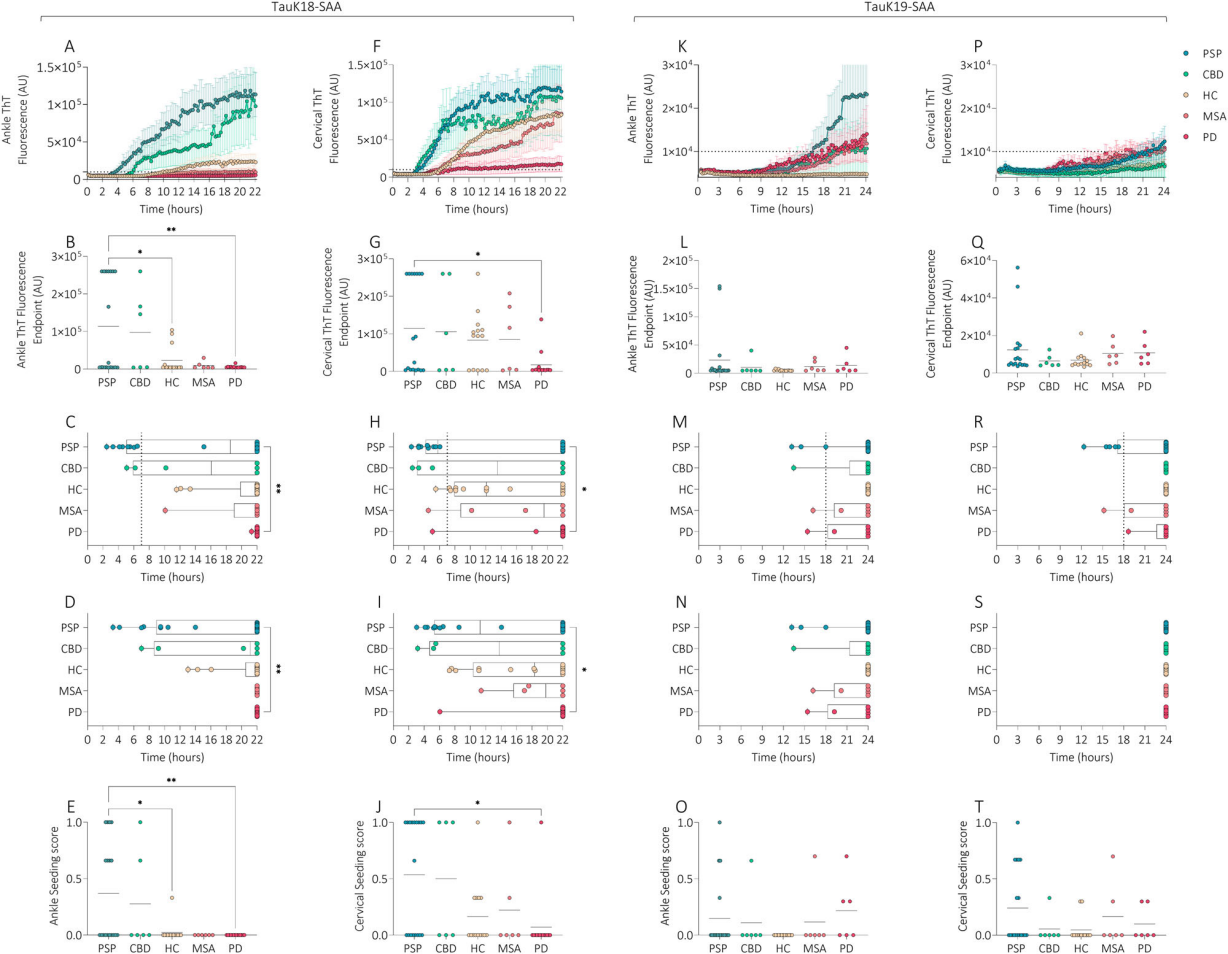
TauK18- and TauK19-SAA analyses at ankle and cervical sites in disease sub-groups. Kinetic curves of TauK18 and TauK19 seeding activity in the ankle (**A**, **K**) and cervical (**F**, **P**) sites, respectively. Each dot represents the mean (±SE) of ThT fluorescence intensity of all samples tested per group against time. The dotted line indicates the selected threshold at 10.000 arbitrary units (AU). **B**, **G**, **L**, **Q** Scatter plot of final ThT fluorescence intensities: dots represent the mean values of the final ThT fluorescence intensity for individual patients and bars the mean of the group. **C**, **H**, **M**, **R** Box and Whisker Plot showing the Lag time defined as the reaction time required to pass the threshold of 10.000 AU. The dotted line shows the selected time points (7 h for TauK18 or 18 h for TauK19) to define the positive and negative subjects. **D**, **I**, **N**, **S** Box and Whisker Plot showing the T50 defined as the reaction time required to reach 50% of the maximum fluorescence detected. **E**, **J**, **O**, **T** Seeding score defined as the number of replicates out of three that cross the threshold of 10.000 AU at 7 h. Each dot represents a patient and bars the mean of the group.

**Fig. 3:**
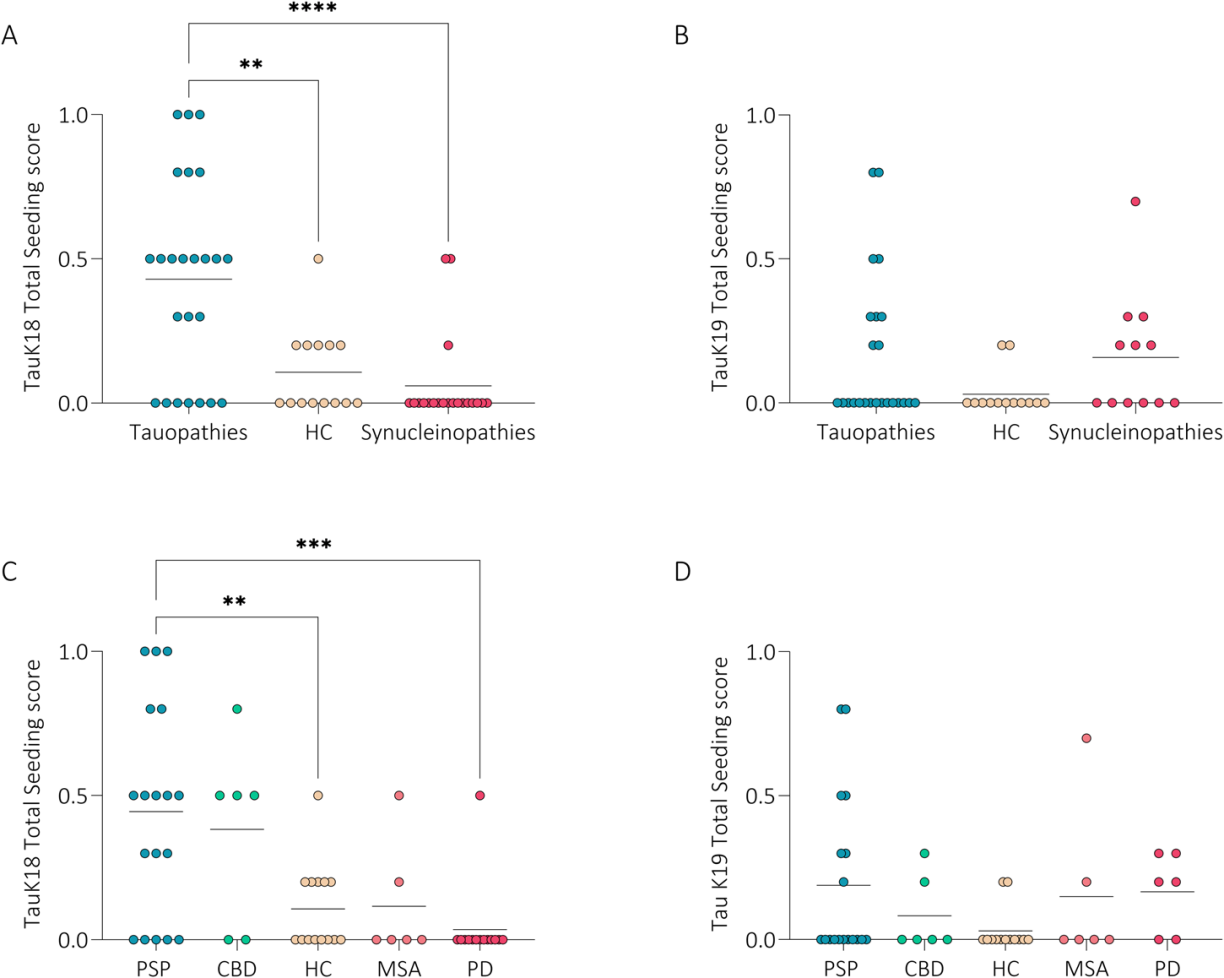
Total seeding score. Seeding score, defined as the number of positive replicates out of three for TauK18 and TauK19, considering the three main groups (**A**, **B**), or subgroups of diseases (**C**, **D**).

TauK18-SAA analysis in subgroups showed similar kinetic patterns in PSP and CBD in cervical area, but a shorter lag time was noted in ankle in PSP. MSA and HC showed higher ThT signal and shorter lag time in cervical than ankle. PSP displayed significantly higher ThT signal, lag time, T50, and seeding score than PD in both locations and higher ThT signal and seeding score vs HC in ankle (Fig. 2A–J). Total seeding score was significantly higher in tauopathies vs HC and synucleinopathies (Fig. 3).

TauK19-SAA showed 3R aggregation in a few sets of samples, and kinetic curves were characterized by lower ThT fluorescence, longer lag time and T50, and reduced seeding score than those observed with TauK18 (Fig. 1K–T). No significant differences were found between groups, even if a steeper curve was observed in ankle in tauopathies. TauK19-SAA showed negative seeding activity in HC in both locations and in CBD in cervical area (Fig. 2K–T).

### Biochemical characterization of SAA end-products

**Fig. 4:**
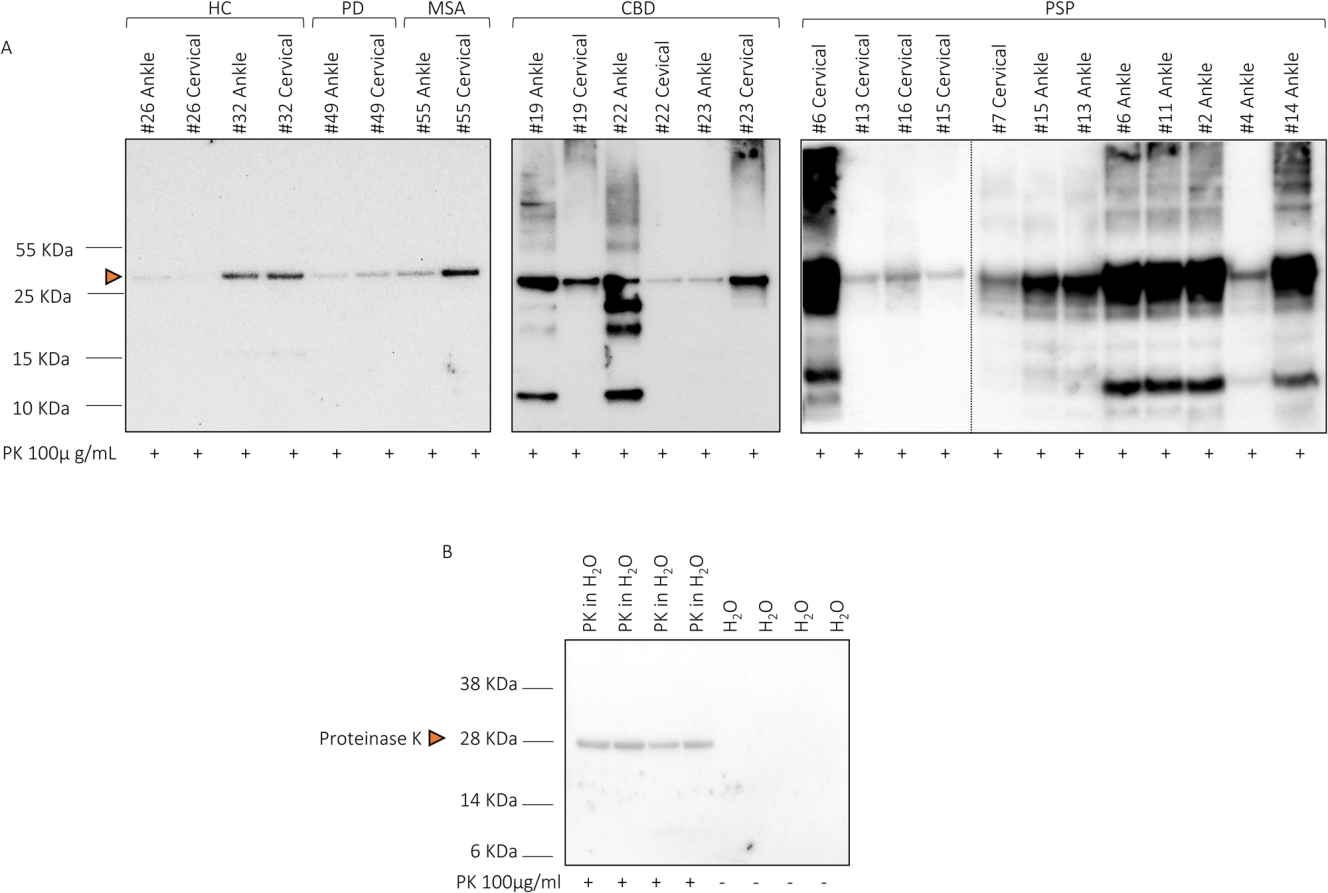
Western blot analysis of TauK18-SAA end-products after digestion with Proteinase K. **A** Band profiles of representative cases in each disease group in ankle and cervical sites are shown. The head arrow indicates that the band at 28 kDa might be partially due to an unspecific signal of PK. **B** Technical control confirming PK unspecific signal at 28 KDa. Experiments were performed three times, and gels were processed in parallel.

SAA end-products obtained from skin lysates with positive TauK18-SAA and from 1HC with negative reaction, as negative control (#26), were analyzed by Western blot after digestion with Proteinase K (PK, Fig. 4). Overall, the samples of HC and alpha-synucleinopathies positive to SAA (#32C, #49C, #55C) did not show bands after PK digestion compared to tauopathies, suggesting that the biochemical properties of the final aggregates significantly differed from those generated by PSP and CBD. In tauopathies, bands migrating between 25 and 10 kDa were observed, while bands with higher molecular weight, above 55 kDa, were observed mainly in samples from ankle with exception of a PSP (#6). Within the same subject, we observed higher resistance to PK digestion in ankle vs cervical skin in both CBD (#19) and PSP in cervical (#13).

### TEM characterization of skin SAA end-products

**Fig. 5:**
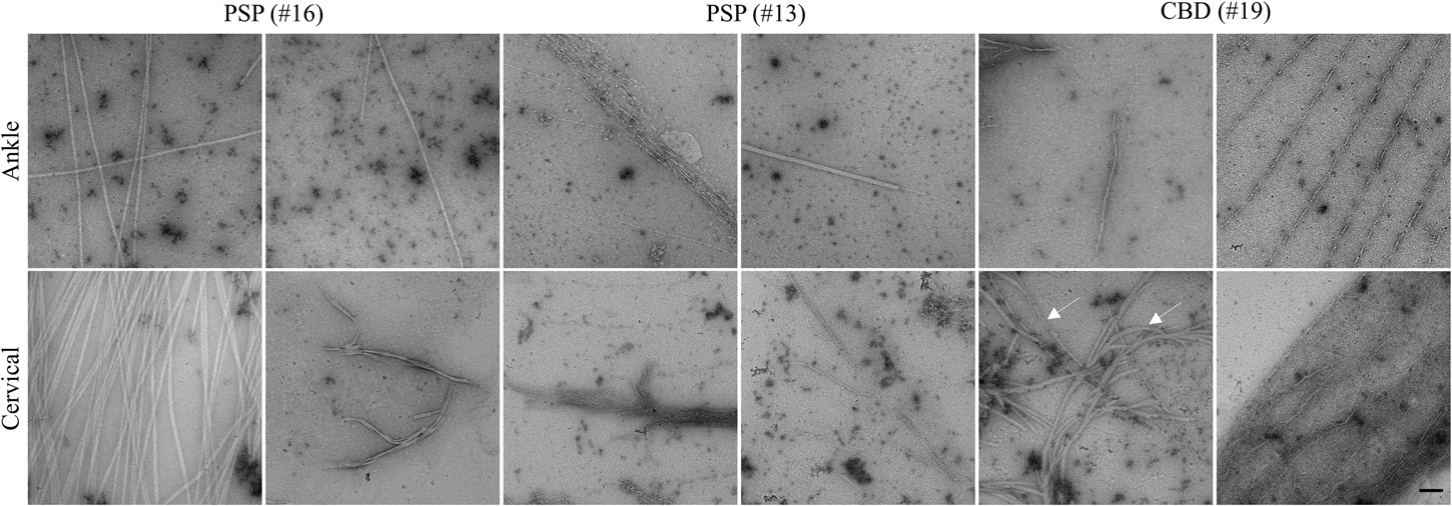
TauK18-SAA end-products. TEM images of TauK18-SAA end-products from ankle and cervical sites in PSP and CBD subjects. Arrows indicate different morphologies of tau fibrils within the same patient and anatomical site. Scale bar 100nm.

Skin TauK18-SAA end-products of 4 PSP, 2CBD, 1HC, 1 PD, and 1MSA, which resulted positive to the assay, were analyzed by transmission electron microscopy (TEM). No fibril-like structure was detectable in HC and alpha-synucleinopathies (Supplementary Fig. 1). In contrast, numerous fibrils with various morphologies were evident in PSP and CBD cases (Fig. 5). In general, we observed a higher amount of single, straight fibrils in samples from ankle biopsies, while curvy and larger caliber-structure were more abundantly present in samples from cervical skin. Further, fibrils with diverse morphology were seen within the same patient and same location (PSP#16, CBD#19).

### Skin TauK18-SAA distinguishes 4R tauopathies with more sensitivity than TauK19-SAA

**Fig. 6:**
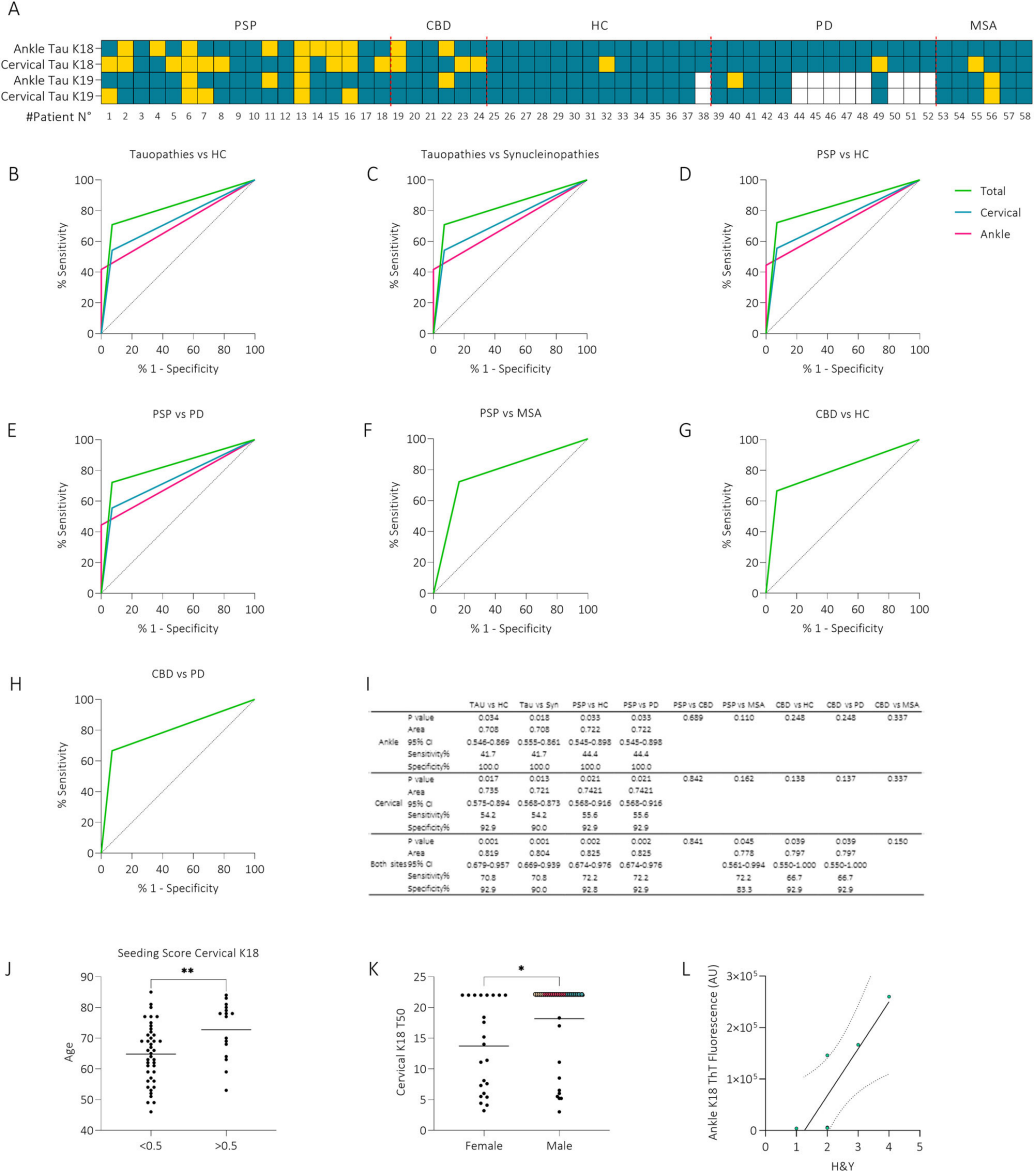
Diagnostic performance and correlation analysis. **A** Heatmap showing positive (yellow) and negative (blue) patients analyzed with TauK18 and TauK19 in ankle and cervical sites. **B**–**H** ROC curves analysis of TauK18 positivity/negativity in ankle (fuchsia), cervical (blue), and both sites (green). The referral line is reported in black. **I** The table provides the asymptotic significance, AUC with 95% CI, sensitivity, and specificity for each comparison. *P*-values < 0.05 were considered significant. **J** Box plot showing differences in age between subjects with seeding scores lower or higher than 0.5. Each dot represents a patient and bars the mean of the group. **K** Box plot showing differences in cervical TauK18 T50 between male and female subjects. Each dot represents a patient and bars the mean of the group. **L** Correlation between ankle TauK18 ThT fluorescence signal and H&Y in CBD (R = 0.865, *P* = 0.026, *n* = 6). The regression line is reported together with its 95% CI (dashed line).

Considering TauK18-SAA and both anatomical sites, 17 out of 24 tauopathies (70%), 1 out of 14 HC (7%), and 2 (1 PD, 1 MSA) out of 20 synucleinopathies (10%) resulted positive. Among tauopathies negative to TauK18-SAA, 2 out of 7 (29%) were possible or suggestive PSP, while only 2 out of 17 (12%) were possible PSP in the group of those positive to the assay, being the majority classified with higher probability level (Table 3). No differences in disease duration or clinical impairment were observed between tauopathy patients, positive or negative, to tauK18-SAA. The 2 alpha-synucleinopathies positive to tauK18-SAA were a deNovo PD and a possible MSA (Table 3). Among tauopathies, 13 were positive in cervical, 10 in ankle, and 6 at both sites, while HC and synucleinopathies resulted positive in cervical site only. Considering both sites, 13 out of 18 (72%) PSP and 4 out of 6 (67%) CBD resulted positive (Fig. 6A).

**Table 3:** Clinical diagnosis and TauK18-SAA results

Patients n°	Clinical diagnosis	TauK18-SAA
#1	Possible PSP-RS	Positive
#2	Probable PSP-P	Positive
#3	Probable PSP-RS	**Negative**
#4	Probable PSP-RS	Positive
#5	Probable PSP-P	Positive
#6	Probable PSP-CBS	Positive
#7	Probable PSP-RS	Positive
#8	Possible PSP-RS	Positive
#9	Possible PSP-CBS	**Negative**
#10	Suggestive of PSP-P	**Negative**
#11	Probable PSP-RS	Positive
#12	Probable PSP-CBS	**Negative**
#13	Probable PSP-PGF	Positive
#14	Probable PSP-RS	Positive
#15	Probable PSP-RS	Positive
#16	Probable PSP-RS	Positive
#17	Probable PSP-P	**Negative**
#18	Probable PSP-RS	Positive
#19	Probable CBD	Positive
#20	Probable CBD	**Negative**
#21	Probable CBD	**Negative**
#22	Probable CBD	Positive
#23	Probable CBD	Positive
#24	Probable CBD	Positive
#39	Definite PD	Negative
#40	Definite PD	Negative
#41	Definite PD	Negative
#42	Definite PD	Negative
#43	Definite PD	Negative
#44	Definite PD	Negative
#45	Definite PD	Negative
#46	Definite PD deNovo	Negative
#47	Definite PD deNovo	Negative
#48	Definite PD deNovo	Negative
#49	Definite PD deNovo	**Positive**
#50	Definite PD deNovo	Negative
#51	Definite PD deNovo	Negative
#52	Definite PD deNovo	Negative
#53	Probable MSA-C	Negative
#54	Possible MSA-P	Negative
#55	Possible MSA-P	**Positive**
#56	Possible MSA-P	Negative
#57	Possible MSA-C	Negative
#58	Possible MSA-P	Negative

In bold, SAA results discordant with the clinical diagnosis. PSP diagnosis according to the MDS criteria^35^; RS = PSP with Richardson’s syndrome, P = PSP Parkinsonism predominant, CBS = PSP with corticobasal syndrome, PGF = PSP with primary gait freezing. Clinical diagnosis of Probable CBD according to diagnostic criteria^36^. Clinical diagnosis of Definitive Idiopathic PD according to diagnostic criteria^37^. MSA diagnosis according to the second consensus diagnostic criteria for MSA^38^; P = MSA with predominant parkinsonism, C = MSA with predominant cerebellar ataxia.

Considering TauK19-SAA and both anatomical sites, 7 out of 24 (29%) tauopathies, 0 out of 13 HC, and 2 (1 PD, 1 MSA) out of 12 (16%) synucleinopathies resulted positive. Among tauopathies, 5 were positive in cervical site, 4 in ankle site, and 2 at both sites; 1PD was positive in ankle and 1 MSA in both locations. Among sub-groups, 6 out of 18 (33%) PSP and 1 out of 6 (17%) CBD resulted positive (Fig. 6A).

Receiver operating characteristics (ROC) curve analysis was performed only for TauK18-SAA and showed the best performance in distinguishing tauopathies vs HC and tauopathies vs synucleinopathies, combining both anatomical sites. Considering sub-groups, the accuracy was slightly higher for PSP than CBD. Moreover, it was possible to discriminate PSP vs MSA patients (Fig. 6B–I).

Further, stratifying all subjects based on seeding activity, those with higher seeding scores were significantly older (Fig. 6J). Considering all subjects, females showed higher TauK18 seeding activity in cervical site measured by T50 (*P* = 0.020), which was confirmed in HC (*P* = 0.022) and synucleinopathies (*P* = 0.040), but not in tauopathies (*P* = 0.413) (Fig. 6K).

In CBD, TauK18-SAA ThT signal in ankle directly correlated with H&Y (Fig. 6L), and the result was confirmed by multivariate linear regression analysis after adjusting for age and sex (*P* = 0.041, B = 0.8).

## Discussion

This work showed that tau-SAA analysis of skin biopsies is feasible and distinguishes living patients with tauopathies from those with synucleinopathies and HC with 73% sensitivity and 93% specificity in concordance with the clinical diagnosis. This result represents an impactful step for developing sensitive and non-invasive biomarkers for tauopathies, whose diagnosis still relies only on clinical signs. While recent advances in PET imaging and sensitive immunoassays of phosphorylated and total tau in plasma and CSF have improved the diagnosis of AD^23^, this is not yet true for tauopathies and synucleinopathies: PET tracers are still developing, and no reliable biomarker in plasma/CSF is ready to use in clinic for tauopathies. Therefore, skin biopsy, which is low cost and minimally invasive, less than a lumbar puncture, and repeatable in time with minimal discomfort for patients^23^, may improve the current clinical management of patients with tauopathies.

We observed in skin a greater sensitivity of TauK18-SAA than TauK19-SAA for PSP and CBD, which are characterized mainly by 4R aggregates in brain, showing that tau seed in peripheral tissues may reflect what has been observed in brain. The expression level of substrates can amplify the pathogenic seed and contribute to the selected vulnerability of different neuronal populations^24,25^, and we previously showed higher concentrations of tau protein and higher levels of 4R tau transcripts in skin lysates of PSP and CBD vs synucleinopathies and HC^22^. In support of these results, another recent study has shown the presence of phosphorylated and 4R deposits in autoptic peripheral nerves, namely cranial and anterior spinal root nerves, of patients with PSP^26^. Nevertheless, few works in literature have explored the diagnostic capacities of 4R substrates in PSP and CBD in brain, and no one in skin. Of note, the kinetic characteristics of skinTauK18-SAA in this study resemble those previously described in post-mortem brains of PSP and CBD in terms of lag time and ThT fluorescence peak, while the seeding activity in skin seems higher compared to the one described in CSF of living patients^11^. However, the use of TauK18CFh in the latter study^11^, a different cysteine-free substrate with mutations at residues 291 and 322 cysteine to serine and a poly-histidine tag, might partially explain the differences.

Cervical skin biopsy showed the highest sensitivity for tauopathies, while ankle biopsy results were more specific and did not show any positivity for TauK18 in both synucleinopathies and HC. In analogy, several studies on pathologic alpha-synuclein in skin biopsies showed higher sensitivity of cervical area in differentiating PD^18,20,27,28^. The cervical area bears the advantage of being anatomically closer to the CNS and brainstem. Another explanation may be related to the physiologically higher innervation density in proximal vs distal skin^29^, with consequently higher amount of tau and higher propensity to seed/aggregate. This might also explain the lower aggregation activity in PD in cervical compared to MSA and HC; indeed, several studies have reported a skin denervation in proximal sites in PD^18,20,30^.

Differences in anatomical regions were also noted in terms of biochemical characterization of TauK18-SAA end-products in PSP and CBD, where higher PK-resistance and more pronounced differences in banding profiles of aggregates generated in ankles were detected than in cervical. This finding and the absence of false positive reactions in ankle may suggest that skin TauK18-SAA from ankle is more specific, even if less sensitive. Of note, the final aggregates in HC and alpha-synucleinopathies positive to the assay showed no PK-resistance, differently from those generated in PSP and CBD. Thus, although these findings are based on a limited number of cases, we observed different biochemical properties of SAA end-products according to disease group, anatomical area, and within the same subject. In line with these results, the morphological analysis of SAA end-products by TEM confirmed diverse structures of fibrils in ankle vs cervical area and within the same subjects. Albeit it is still debated if SAA end-products can mirror the pathological aggregates in brain, this finding supports the concept that a different cellular environment might dictate a different seed in the same patient.

Our data reveal a correlation between skin tau seeding activity and age in all subjects; this is an intriguing finding, as aging is a major risk factor for neurodegenerative diseases. However, this result might be influenced by patients being older than HC. Therefore, further studies are needed to determine whether aging is independently associated with a higher tau seeding propensity in skin. Female subjects presented a significantly higher tau seeding activity than males in HC and synucleinopathies but surprisingly not in tauopathies. This finding aligns with the observation that late middle-aged women, compared with men, show higher tau burden, independently of amyloid β by tau and amyloid Positron Emission Tomography (PET) analysis^31^. We found a direct correlation of tau seeding activity with H&Y scale in CBD. However, no other correlation with disease gravity was detected in PSP; this might be due to the use of MMSE, MOCA, and MS-UPDRS III, which are less specific and sensitive scales for tauopathies. This may underscore the need for more comprehensive neuropsychological assessments targeting frontal-executive domains^32^, as well as the use of disease-specific motor and functional validated scales, such as the PSP Rating Scale (PSPRS)^33^.

Alpha-synuclein-SAA in skin has shown high sensitivity and specificity in detecting PD also at prodromal stages like in RBD patients^34^. Our results on TauK18-SAA in tauopathies open the alluring possibility of integrating both analyses in a minimally-invasive skin biopsy, allowing optimal diagnostic accuracy and assessment of co-pathologies in-vivo in neurodegenerative diseases. In fact, the presence of co-pathologies in neurodegenerative diseases is frequently shown in brain autoptic studies, it increases with age and is probably in part responsible for the heterogeneity of clinical phenotypes. In this regard, we cannot exclude a possible concomitant AD pathology in our cohort, especially in CBD patients where cognitive impairment is a frequent feature since only a few patients underwent AD biomarkers analysis in CSF. This is linked to the major limitation of the present study, which is the lack of autoptic pathological confirmation of the clinical diagnosis and assessment of co-pathologies. Other limitations include the small number of CBD and MSA and the absence of patients with 3R tauopathies and AD. In addition, we acknowledge that even if HC were enrolled only when neurological diseases were excluded by medical history, no neurocognitive tests or other diagnostic investigations were performed to rule out possible early and subclinical neurodegenerative diseases. In contrast, the strengths of the study lie in the evaluation of a prospective cohort of living patients with neurodegenerative diseases, including a group of sex-matched HC, the analysis of multiple skin biopsies at the same time point, and the exclusion of other concomitant systemic disorders.

In conclusion, Tau-SAA in skin may contribute to a more accurate clinical diagnosis of tauopathies, and an optimized version of the assay can be extended to the analysis of skin samples in AD and for monitoring disease progression and efficacy of disease-modifying therapies.

## Methods

### Subjects’ recruitment

Patients were consecutively recruited from the movement disorders outpatient clinic at NSI Lugano as part of the NSIPD001 case-control study from 09.2018 to 09.2023, and from the movement disorders unit at Hospital de la Santa Creu i Sant Pau, Barcelona, as part of the IIBSP-PAR-2022-98 study from 12.2022 to 08.2023. HC, without any known neurological disease and systemic disease, were recruited among hospital staff and patients’ partners.

Inclusion criteria for PSP^35^, CBD^36^, PD^37^, and MSA^38^ were based on published diagnostic criteria. PD group included deNovo patients, defined as subjects with criteria for a definite diagnosis of PD, <2 years since the diagnosis, and no dopaminergic treatment at the moment of enrollment.

Exclusion criteria for all participants were: family history of neurodegenerative disorders and significant comorbidities such as diabetes, renal failure, thyroid pathology, vitamin B12 deficiency, HIV infection, syphilis, coagulopathy, acute and chronic inflammatory diseases, and tumors.

This study was approved by the Canton Ticino Ethical Committee (CE TI 2895) and performed in line with the principles of the Declaration of Helsinki. All subjects were included according to the study protocol and gave written informed consent to the study.

### Clinical assessment

Patients underwent clinical assessment by: H&Y^39^ scale, MDS-UPDRS^40^, MMSE^41^, MoCA^42^, Beck Depression Inventory-II (BDI-II)^43^, Composite Autonomic Symptom Score 31 (COMPASS-31)^44^, RBDSQ^45^. Patients with tauopathies also underwent Frontal Assessment Battery (FAB)^46^ scale. LEDD^47^ was calculated for all patients. As part of the diagnostic work-up, 5 patients with tauopathies (4 PSP, 1CBD) underwent CSF β amyloid 42/40 ratio, total, and phospho-tau analysis, which resulted within normal ranges. All patients underwent brain MRI imaging that was rated negative for atrophy patterns suggestive of AD. Further, we performed α-synuclein-SAA in cervical skin biopsy in the group of alpha-synucleinopathies to support their clinical diagnosis (Supplementary Fig. 2). It resulted positive in 17/20 patients with synucleinopathies (11/14 PD and 6/6 MSA) and 1/6 HC (sensitivity 85%, specificity 83%) in line with previously published reports^48^^,^^49^]. However, PD patients who tested negative at α-synuclein-SAA had enough clinical and supportive criteria for the diagnosis of PD, and the results may be related to the limitation of the assay.

### Skin biopsy collection and skin homogenate

On the more clinically affected side, a 3-mm-diameter punch skin biopsy was performed in the neck at the C8 dermatomal level (cervical) and the distal leg 10 cm above the lateral malleolus (ankle)^18,20,22^. The biopsies were stored at −80 °C until use.

Frozen skin samples were washed in ice-cold PBS 1X (Sigma) at least three times to remove blood and homogenized at 1% in lysis buffer in tubes containing zirconia beads (0.7 mm, BioSpec) to facilitate the tissue disruption. Homogenization was performed with TissueLyser LT (Qiagen): 1’lysis/1’ice ×5 times. Lysates were used for SAA analyses.

### Tau peptide expression and purification

The expression and purification of human Tau proteins were performed as previously described^50^. pET11a plasmids encoding for the human TauK18 and TauK19 constructs were transformed in E. coli BL21 (DE3) cells (New England Biolabs). Cells were grown in 2 L of Luria-Bertani medium, and protein expression was induced by adding 0.8 mM Isopropyl β-D-1-thiogalactopyranoside (IPTG) (PanReac Applichem). Cell lysates were subjected to a precipitation step by boiling the homogenates for 20 min before two subsequent purification steps by cation exchange and size-exclusion chromatography. Purified proteins were analyzed by sodium dodecyl sulfate-polyacrylamide gel electrophoresis (SDS-PAGE), lyophilized, and stored at −80 °C.

### Seed amplification assay

2 μL of skin lysate were added to the reaction mix containing 0.1 mg/mL of TauK18, TauK19, or recombinant α-Synuclein (rPeptide) and analyzed in triplicate in a 96-well optical flat bottom plate (Thermo Fisher Scientific) with one glass bead per well (3-mm, Sigma). The plates were subjected to alternative cycles of shaking (600 rpm orbital, 1 minute) and incubation (14 min.) at 42 °C in a CLARIOstar Plus (BMG LABTECH) plate reader. The ThT fluorescence intensities, expressed as arbitrary units (AU), were taken every 14 min using 450 ± 10nm (excitation) and 480 ± 10nm (emission) wavelengths, with a bottom read. The assay was performed by an operator blind to the clinical diagnosis.

### Seed amplification assay data analysis

For TauK18 and TauK19, reactions were considered positive if ThT fluorescence exceeded the threshold, set at 10000 AU, chosen as the one that best distinguished samples of PSP/CBD from the others. The cut-off time was set at 7 h for TauK18 and 18 h for TauK19. For α-Synuclein, the ThT threshold was set at 1000 AU, and the cut-off time at 18 h, chosen as the one that best distinguished PD/MSA from HC.

A sample was considered positive if at least 2 out of 3 replicate wells exceeded this threshold. Tau-SAA end-point fluorescence, lag time, and T50 were analyzed. Seeding score was calculated as the ratio of positive replicates out of 3 in ankle and cervical sites^49^. Total seeding score was calculated as the ratio of positive replicates in both ankle and cervical sites.

### Proteinase K digestion and Western Blot

20 μl of final TauK18-SAA products were treated with 100 μg/mL of PK (Invitrogen) for 1 hour at 37 °C under shaking (500 rpm). Digestion was stopped directly by adding LDS-PAGE loading buffer (Bolt™ LDS Sample Buffer and Dithiothreitol (DTT), Thermo Scientific) at 100 °C for 10 min.

Samples were loaded into 12% Bolt Bis-Tris Plus gels (Invitrogen), and proteins were separated using SDS-PAGE and then transferred onto polyvinylidene difluoride membranes (PVDF Immobilon-P, Millipore). After the incubation with 5% (weight/volume) non-fat dry milk (Santa Cruz) for 1 h at room temperature under shaking, the PVDF membrane was incubated overnight at 4 °C under shaking with RD4 antibody (4-repeat isoform tau mouse monoclonal antibody, clone 1E1/A6, ThermoFischer Scientific) to visualize final SAA products. Finally, membranes were incubated with anti-mouse secondary antibody conjugated with horseradish peroxidase (GE) and developed with chemiluminescent system (ECL Prime, GE Healthcare Amersham). Reactions were visualized using Chemidoc MP Imaging System (Biorad).

### Transmission electron microscopy

Two µL of skin TauK18-SAA end-products were dropped on a carbon film 300 mesh grid previously glow discharged. After 5 min, the excess sample was removed by filter paper, and the grid was stained with Uranyl Acetate Replacement (UAR, Electron Microscopy Sciences) diluted 1:4 in milli-Q water. After 2 min, the solution was removed, and the grid was air-dried for 30 min before the analyses. Representative images were taken using a Talos L120C electron microscope operating at 120 kV with a 4 K × 4 K Ceta CMOS Camera (FEI, Thermo Fisher Scientific).

### Statistical analysis

Statistical analysis was performed using IBM SPSS Statistics 26.0. Kolmogorov–Smirnov test assessed the distribution of variables. 1-way ANOVA test with post-hoc Bonferroni’s test for multiple comparisons was used for normally distributed variables, expressed as mean ± standard deviation (SD). Kruskal-Wallis test was used for non-normally distributed variables, expressed as medians and interquartile range. χ² or Fisher’s exact tests were used for categorical variables, defined as a percentage (%). ROC curve analysis was used to evaluate the area under the curve (AUC) and compare diagnostic performances. In addition to AUC, P value, 95% confidence interval (CI), sensitivity, and specificity were reported for each ROC analysis. Correlations were evaluated by Pearson’s R test and regression curve analysis; correlations were considered strong for R between |1.0| and |0.5|, moderate between |0.5| and |0.3|, weak between |0.3 and |0.1|. Multivariate linear regression analysis, adjusted for age and sex, was performed to corroborate the significant correlations. A *P*-value lower than 0.05 was considered significant. The sample size of the study was dictated by the number of skin biopsies available. For clinical variables, missing data were not replaced, while tau-SAA data were available for all subjects.

### Supplementary information

SUPPLEMENTAL MATERIAL

## Data Availability

The raw data supporting this article’s findings are available to the corresponding author upon request.
