# Supplementary material for: Tau seeding activity in skin biopsy differentiates tauopathies from synucleinopathies
Source: NPJ Parkinsons Dis. 2024 Jun 15;10:116. doi: 10.1038/s41531-024-00728-9 (PMC11180195; doi:10.1038/s41531-024-00728-9)
Supplement: Supplementary file 1 — SUPPLEMENTAL MATERIAL [file 41531_2024_728_MOESM1_ESM.pdf]

SUPPLEMENTARY MATERIAL

Tau seeding activity in skin biopsy differentiates tauopathies from synucleinopathies

Ilaria Linda Dellarole M.Sc.[1*], Elena Vacchi Ph.D.[2,3*], Inigo Ruiz-Barrio M.D.[4], Sandra Pinton M.Sc.[2,5], Andrea Raimondi, Ph.D.[6], Stefania Rossi M.Sc.[5], Sara Morandi M.D.[5], Giovanni Bianco M.D.[5], Merve Begum Bacinoglu M.Sc.[1], Annalisa Lombardo M.Sc.[1], Luigi Celauro Ph.D.[7], Claudio Staedler M.D.[5], Salvatore Galati M.D. Ph.D.[3,5], Javier Pagonabarraga M.D. Ph.D.[4], Jaime Kulisevsky M.D. Ph.D.[4], Giuseppe Legname Ph.D.[7], Claudio Gobbi M.D.[3,5,8], Alain Kaelin-Lang MD Ph.D.[3,5,9], Fabio Moda Ph.D.[1#], Giorgia Melli M.D. Ph.D.[2,3,5#]

Author affiliations:

1. Division of Neurology 5 and Neuropathology, Fondazione IRCCS Istituto Neurologico Carlo Besta, Milan, Italy.
2. Neurodegenerative Diseases Group, Laboratory for Translational Research, Ente Ospedaliero Cantonale, Bellinzona, Switzerland.
3. Faculty of Biomedical Sciences, Università della Svizzera Italiana, Lugano, Switzerland.
4. Movement Disorders Unit, Neurology Department, Hospital de la Santa Creu i Sant Pau, Barcelona, Spain.
5. Neurology Department, Neurocenter of Southern Switzerland, Ente Ospedaliero Cantonale, Lugano, Switzerland.
6. Institute for Research in Biomedicine, Università della Svizzera italiana, Bellinzona, Switzerland
7. Laboratory of Prion Biology, Department of Neuroscience, Scuola Internazionale Superiore Di Studi Avanzati (SISSA), Trieste, Italy
8. Department of Neurology, University Hospital of Basel, Basel, Switzerland
9. Department of Neurology, Inselspital, Bern University Hospital, University of Bern, Bern, Switzerland

*Equally contributed to the paper

Co-last authors

Correspondence to: Prof. Giorgia Melli Neurodegenerative Diseases Group, Laboratory for Translational Research, Ente Ospedaliero Cantonale, via Francesco Chiesa 5, 6500 Bellinzona, Switzerland E-mail: giorgia.melli@eoc.ch Tel: +41 (0)91 8116535; Fax: +41 (0)91 8116915

Summary:

Supplementary Table 1

Supplementary Table 2

Supplementary Figure 1

Supplementary Figure 2

Supplementary Table 1

	Variable	HC (n=14)	TAU (n=24)	SYN (n=20)	Overall P-Value	TAU vs HC	SYN vs HC	TAU vs SYN
TauK18	ThT End fluorescence Ankle	23114 ± 9794	109595 ± 24221	6862 ± 1385	**0.000**	**0.004**	0.560	**0.000**
	ThT End fluorescence Cervical	83394 ± 20072	112111 ± 24601	37739 ± 14526	**0.039**	0.638	0.352	**0.030**
	Lag Time (h) Ankle	19.9 ± 1.1	14.1 ± 1.7	21.4 ± 0.6	**0.000**	**0.015**	0.771	**0.001**
	Lag Time (h) Cervical	13.9 ± 1.8	12.3 ± 1.8	19.3 ± 1.3	**0.011**	0.803	0.112	**0.009**
	T50 (h) Ankle	20.4 ± 0.8	16.2 ± 1.5	22.0 ± 0.0	**0.000**	**0.034**	0.615	**0.001**
	T50 (h) Cervical	16.4 ± 1.6	12.3 ± 1.7	20.2 ±0.9	**0.005**	0.364	0.236	**0.003**
	Seeding Score Ankle	0.02 ± 0.02	0.34 ± 0.08	0.0 ± 0.0	**0.000**	**0.004**	0.969	**0.001**
	Seeding Score Cervical	0.16 ± 0.07	0.52 ± 0.10	0.11 ± 0.06	**0.002**	**0.024**	0.932	**0.003**
	Seeding Score Total	0.11 ± 0.03	0.42 ± 0.07	0.06 ± 0.03	**0.000**	**0.001**	0.855	**0.000**
TauK19	ThT End fluorescence Ankle	5086 ± 357	20051 ± 8515	12912 ± 3564	0.356	0.328	0.794	0.782
	ThT End fluorescence Cervical	6899 ± 1285	10894 ± 2664	10625 ± 1678	0.483	0.478	0.620	0.996
	Lag Time (h) Ankle	24.0 ± 0.0	21.9 ± 0.9	22.5 ± 0.7	0.195	0.311	0.203	0.859
	Lag Time (h) Cervical	24.0 ± 0.0	22.1 ± 0.7	22.5 ± 0.8	0.204	0.184	0.441	0.933
	T50 (h) Ankle	24.0 ± 0.0	21.9 ± 0.9	22.5 ± 0.7	0.195	0.311	0.203	0.859
	T50 (h) Cervical	24.0 ± 0.0	24.0 ± 0.0	24.0 ± 0.0	1.000	1.000	1.000	1.000
	Seeding Score Ankle	0.00 ± 0.00	0.12 ± 0.6	0.16 ± 0.7	0.178	0.237	0.218	0.946
	Seeding Score Cervical	0.04 ± 0.03	0.20 ± 0.6	0.13 ± 0.06	0.264	0.235	0.665	0805
	Seeding Score Total	0.03 ± 0.02	0.16 ± 0.05	0.15 ± 0.06	0.173	0.177	0.295	0.998

Kinetic parameters of TauK18 and TauK19 seeding activity at ankle and cervical in main groups. Variables are reported as mean ± SE. A $P<0.05$ was considered significant and shown in bold.

Supplementary Table 2

	Variable	HC (n=14)	PSP (n=18)	CBD (n=6)	PD (n=14)	MSA (n=6)	Overall P-value	PSP vs HC	CBD vs HC	PD vs HC	MSA vs HC	PSP vs CBD	PSP vs PD	PSP vs MSA	CBD vs PD	CBD vs MSA	PD vs MSA
TauK18	ThT End fluor. Ankle	23114 ± 9794	113535 ± 29659	97773 ±44513	5632 ±816	9733 ± 4233	**0.002**	**0.022**	0.334	0.978	0.997	0.993	**0.004**	0.063	0.150	0.337	0.999
	ThT End fluor. Cervical	83394 ± 20072	114312 ± 28864	105506 ± 51244	17494 ± 9939	84976 ± 37892	0.074	0.886	0.988	0.354	0.999	0.999	**0.042**	0.936	0.320	0.995	0.585
	Lag Time (h) Ankle	19.9 ± 1.1	13.9 ± 2.1	14.6 ± 3.3	21.9 ± 0.1	21.0 ± 1.9	**0.004**	0.057	0.384	0.903	0.999	0.999	**0.004**	0.228	0.110	0.537	0.965
	Lag Time (h) Cervical	13.9 ± 1.8	12.2 ± 2.1	12.8 ± 4.1	20.5 ± 1.2	16.3 ± 3.0	**0.037**	0.965	0.998	0.157	0.966	0.999	**0.024**	0.772	0.238	0.929	0.718
	T50 (h) Ankle	20.4 ± 0.8	15.8 ± 1.7	17.7 ± 2.8	22.0 ± 0.0	22.0 ± 0.0	**0.006**	0.095	0.656	0.911	0.936	0.984	**0.009**	0.082	0.270	0.437	0.999
	T50 (h) Cervical	16.4 ± 1.6	13.3 ± 1.9	13.3 ± 3.8	20.8 ± 1.1	18.7 ± 1.7	**0.028**	0.704	0.888	0.413	0.956	0.999	**0.022**	0.451	0.169	0.653	0.963
	Seeding Score Ankle	0.02 ± 0.02	0.37 ± 0.10	0.28 ± 0.18	0.00 ± 0.00	0.00 ± 0.00	**0.002**	**0.012**	0.380	0.999	0.999	0.959	**0.006**	0.063	0.292	0.461	0.999
	Seeding Score Cervical	0.16 ± 0.07	0.53 ± 0.11	0.50 ± 0.22	0.07 ± 0.07	0.22 ± 0.16	**0.013**	0.086	0.439	0.970	0.998	0.999	**0.016**	0.464	0.200	0.751	0.939
	Seeding Score Total	0.11 ± 0.03	0.44 ± 0.08	0.38 ± 0.13	0.04 ± 0.04	0.12 ± 0.08	**0.000**	**0.005**	0.192	0.946	0.999	0.986	**0.000**	0.065	0.055	0.383	0.966
TauK19	ThT End fluor. Ankle	5086 ± 357	23183 ± 11183	10657 ± 5913	14017 ± 6521	11806 ± 3967	0.591	0.488	0.995	0.975	0.992	0.906	0.968	0.932	0.999	0.999	0.999
	ThT End fluor. Cervical	6899 ± 1285	12373 ± 3479	6456 ± 1363	10850 ± 2652	10400 ± 2311	0.555	0.564	0.999	0.928	0.953	0.718	0.997	0.993	0.940	0.958	0.999
	Lag Time (h) Ankle	24.0 ± 0.0	22.5 ± 0.8	22.2 ± 1.8	21.8 ± 1.5	22.1 ± 0.8	0.517	0.689	0.776	0.588	0.708	0.999	0.939	0.997	0.998	0.999	0.999
	Lag Time (h) Cervical	24.0 ± 0.0	21.7 ± 0.9	24.0 ± 0.0	23.1 ± 0.9	21.7 ± 1.5	0.154	0.187	0.999	0.970	0.497	0.4323	0.822	0.999	0.983	0.644	0.915
	T50 (h) Ankle	24.0 ± 0.0	22.5 ± 0.8	22.2 ± 1.8	21.8 ± 1.5	22.1 ± 0.8	0.517	0.689	0.776	0.588	0.708	0.999	0.939	0.997	0.998	0.999	0.999
	T50 (h) Cervical	24.0 ± 0.0	24.0 ± 0.0	24.0 ± 0.0	24.0 ± 0.0	24.0 ± 0.0	1.000	1.000	1.000	1.000	1.000	1.000	1.000	1.000	1.000	1.000	1.000
	Seeding Score Ankle	0.00 ± 0.00	0.15 ± 0.07	0.11 ± 0.11	0.22 ± 0.11	0.12 ± 0.12	0.394	0.418	0.894	0.382	0.894	0.997	0.970	0.997	0.937	0.999	0.937
	Seeding Score Cervical	0.04 ± 0.03	0.24 ± 0.08	0.05 ± 0.05	0.10 ± 0.06	0.17 ± 0.11	0.741	0.514	0.999	0.982	0.982	0.780	0.964	0.964	0.994	0.994	0.999
	Seeding Score Total	0.03 ± 0.02	0.19 ± 0.06	0.08 ± 0.05	0.16 ± 0.05	0.15 ± 0.11	0.336	0.266	0.987	0.689	0.788	0.830	0.999	0.995	0.960	0.982	0.999

Kinetic parameters of TauK18 and TauK19 seeding activity at ankle and cervical sites in disease sub-groups. Variables are reported as mean ± SE. A $P<0.05$ was considered significant and shown in bold.

Supplementary Figure 1

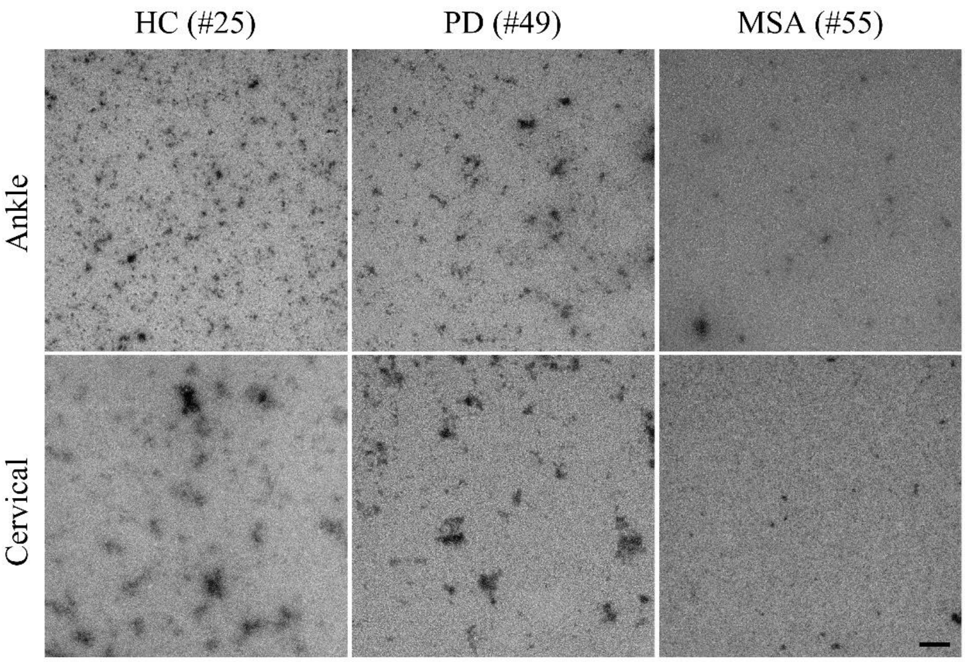

TEM images of TauK18-SAA end-products from ankle and cervical sites from HC, PD, and MSA subjects. Scale bar 100nm.

Supplementary Figure 2

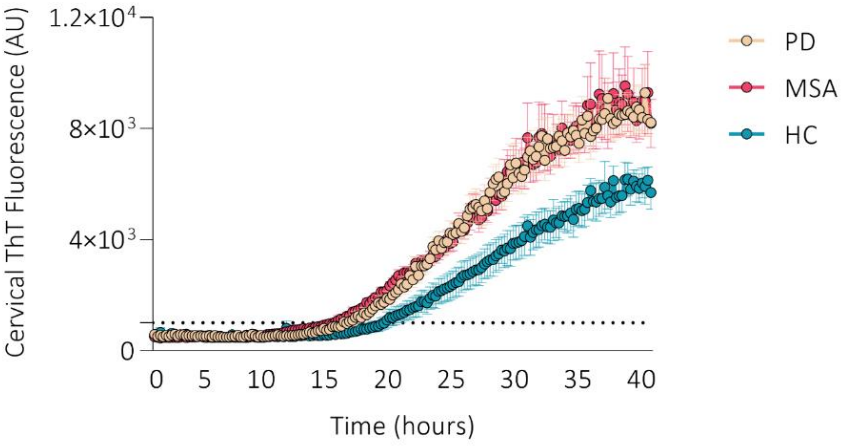

Kinetic curves of Alpha-Synuclein seeding activity in cervical sites. Each dot represents the mean (±SE) of the ThT fluorescence intensity of all samples tested per group against time.
